# Transformational giftedness and sustainability: the role of critical thinking and self-regulation in sustainable-living awareness

**DOI:** 10.3389/fpsyg.2026.1829418

**Published:** 2026-06-22

**Authors:** Zekai Ayık, Muhammet Davut Gül, Mehmet Emin Usta

**Affiliations:** 1 Special Education, Harran University, Şanlıurfa, Türkiye; 2 Special Education, Tokat Gaziosmanpasa University, Tokat, Türkiye; 3 Educational Sciences, Sakarya University, Sakarya, Türkiye

**Keywords:** critical thinking, gifted education, self-regulation, sustainable development goals, transformational giftedness

## Abstract

This study investigated the role of transformational giftedness in fostering sustainability-related skills among middle school students, focusing on critical thinking, self-regulation, and sustainable-living awareness. Grounded in Sternberg’s transformational giftedness concept, the research explored how advanced cognitive and motivational capacities transform into socially meaningful outcomes. A comparative design involved 266 students (118 gifted attending Science and Arts Centers and 148 non-gifted from public schools). Using validated instruments and multiple regression analyses, the study found that gifted students demonstrated significantly higher levels of critical thinking, self-regulation, and sustainable-living awareness. Importantly, both critical thinking and self-regulation predicted sustainable-living awareness in gifted students, while self-regulation showed only marginal predictive power in the non-gifted group. These results suggest transformational giftedness can drive sustainable behavior and that integrating education for sustainable development into gifted education is crucial. Future research should explore longitudinal and cross-cultural pathways to better understand how gifted students contribute to global sustainability goals.

## 1 Introduction

The twenty-first century presents humanity with escalating global challenges such as climate change, inequality, resource depletion, and moral fragmentation, which threaten the sustainability of a livable world (Agbedahin, 2019; Mishra et al., 2023; Yumnam et al., 2024). Such complex and interdependent issues cannot be solved solely through technological innovation or economic reform; they demand profound transformations in human thinking, values, and action. In this respect, education, as a central socializing institution, holds the capacity to foster reflective judgment, moral responsibility, and self-regulated action—psychological foundations of sustainable-living in front of these challenges (Cebrián et al., 2020; Fischer et al., 2022).

Given the above, the United Nations’ 2030 Agenda for Sustainable Development—and the Sustainable Development Goals (SDGs) outlined therein—underscore the need to cultivate individuals capable of critical reflection, ethical decision-making, and proactive engagement in sustainability challenges (UN, 2015; UNESCO, 2017). The education for Sustainable Development (ESD) concept was created as a response to this concern and it operationalizes this vision by engaging key competencies and skills such as systems thinking, anticipatory reasoning, and normative evaluation (Fischer et al., 2022; Wiek et al., 2011). These competencies may enable students to analyze complex socio-environmental systems, envision future scenarios, and assess ethical implications—capacities essential for sustainable transformation. However, achieving the SDGs requires more than mass participation; it also relies on identifying and empowering individuals with the potential to be effective leaders and contribute to social transformation (Finnveden et al., 2020). As such, these individuals, who combine high-level reasoning, creativity, and moral commitment, may act as catalysts of change and achieve the SDGs. In this regard, gifted students, by virtue of their advanced cognitive and motivational potentials (Subotnik et al., 2011), constitute a promising group whose development could accelerate collective progress toward sustainability. Yet, the psychological and social mechanisms through which giftedness translates into sustainable-living awareness and action remain insufficiently understood.

### 1.1 Transformational giftedness: from cognitive excellence to ethical leadership

Traditional one-dimensional and trait-based conceptions of giftedness focused on superior cognitive ability, but contemporary perspectives emphasize multidimensional development that includes psychosocial, motivational, and moral components (Mei et al., 2021; Subotnik et al., 2011), which are dispositions and malleable through *bona fide* educational provisions (Sak, 2023). For example, the Transformational Giftedness Model (Sternberg, 2020; Sternberg et al., 2021) was a major paradigm shift by redefining giftedness as the ethical application of ability to improve the world to be a better place. This model distinguishes between potential giftedness—the input components of cognitive, creative, and motivational capacities—and transformational giftedness—the output manifested as prosocial, civic, and ethical contributions. True giftedness, according to Sternberg (2024a), “is not merely what one knows or can do, but what one chooses to do for the benefit of others.”

Sternberg’s framework integrates his earlier triarchic theory (Sternberg, 2005a) and the WICS model (Sternberg, 2005b), merging analytical, creative, and ethical intelligence into a unified conception of wise action. This synthesis parallels transformational leadership theory (Bass and Riggio, 2005), wherein personal insight and moral conviction drive socially constructive and contributing behavior. In this respect, the transformational gifted individual is seen as a person who embodied “active concerned citizenship” and “ethical leadership” which are the concepts central to sustainable societies. This ethical-developmental view complements humanistic and ecological models that highlight the dynamic interaction among personal ability, psychosocial skills, and environment (Preckel et al., 2020). Subotnik et al. (2011) argue that psychosocial variables—such as self-regulation, perseverance, and moral purpose—are catalysts converting high potential into real achievement. Similarly, Kuusisto and Tirri (2015) demonstrate that gifted adolescents display advanced moral reasoning and prosocial orientation, while Shavinina (2012) note that innovative capacity emerges when cognitive and affective domains interact synergistically.

From this perspective, giftedness is developmental rather than static; it represents a potential that matures through education, social engagement, and moral responsibility. Sternberg (2024b) argues that education should help gifted students progress from potential excellence to transformational wisdom, equipping them not only to think well but also to act well. This socio-moral dimension directly connects gifted education with sustainability education, whose central aim is to cultivate ethical reasoning, self-reflection, and sustainable action. Empirical studies support this link, showing that gifted students often demonstrate greater environmental awareness (Bakar et al., 2018; Tolppanen et al., 2023), moral concern (Gibson and Landwehr-Brown, 2009), and conceptual understanding of socio-scientific issues (Coştu, 2025; Mutlu and Nacaroğlu, 2019). These findings highlight the need for differentiated educational provisions that address gifted students’ distinctive cognitive and higher-order thinking profiles.

Recent research further suggests that constructivist, participatory, and differentiated learning environments can support gifted students’ deeper understanding of sustainability-related issues. For example, interdisciplinary and game-based sustainability education has been shown to foster more systemic and solution-oriented interpretations of environmental and social problems among gifted students (Bezen and Tanriöver, 2026; Kaynar and Kurnaz, 2024). However, research also reveals a “knowledge–action gap”: gifted students may understand environmental issues more deeply than their peers but may not always translate such awareness into observable behavior (Mutlu & Nacaroğlu, 2019). Sternberg’s T-ACCEL model addresses this gap by identifying critical thinking and self-regulation as psychological catalysts that may help transform gifted potential into socially meaningful and sustainability-oriented action (Sternberg, 2024b).

### 1.2 Critical thinking and self-regulation as mechanisms of transformational development

Critical thinking and self-regulation form the cognitive and metacognitive infrastructure of wise, ethical, and sustainable action. In Sternberg (2024b) model, they represent the cognitive and psychosocial catalysts that transform gifted potential into socially meaningful outcomes. Critical thinking involves reasoning pertinent to a specific discipline, reflection, and evaluation in pursuit of justified belief or action (Ennis, 1996; Facione et al., 1995). It combines analytical skills with dispositional virtues such as open-mindedness, humility, and fairness (Bailin et al., 1999; Paul and Elder, 2006). Within sustainability education, it may enable students to navigate moral issues, evaluate competing claims, and envision ethically responsible solutions. Sternberg (2024b) interprets critical thinking as a component of wisdom-based intelligence—the ability to integrate analytical, creative, and ethical reasoning. For gifted students, this capacity supports not only high-level cognition but also ethical discernment, bridging intellect and moral responsibility.

Self-regulation, in turn, refers to the metacognitive and motivational control of one’s thoughts, emotions, and actions to achieve long-term goals (Zimmerman, 1986). It consists of goal setting, self-monitoring, strategic adjustment, and persistence (Zimmerman and Schunk, 2011). As such, it can be seen as an individuals’ ability to plan, monitor, and control cognitive and behavioral processes in pursuit of personally meaningful goals. In this sense, self-regulation may be positioned as the moral discipline: the internal system that governs how intelligence is used. In his model, self-regulation ensures that cognitive strength aligns with ethical purpose and societal good. This resonates with Subotnik et al.’s (2011) view that psychosocial skills serve as the “engine” driving talent development and ethical performance.

The relationship between these constructs is synergistic. Critical thinking provides evaluative insight, while self-regulation sustains goal-directed moral behavior. Without critical thinking, self-regulation risks being misdirected; without self-regulation, critical thinking remains inert. Previous research has highlighted the interconnected nature of higher-order cognitive processes such as metacognitive awareness, self-regulation, and critical thinking in gifted and adolescent students. For example, Boran and Karakuş (2022) found that critical thinking disposition fully mediated the relationship between metacognitive awareness and perceived problem-solving skills among gifted students, while Kuloğlu and Orhan (2024) reported that critical thinking partially mediated the relationship between self-regulation and academic motivation among secondary school students. Together, these findings suggest that critical thinking and self-regulatory processes may function as complementary mechanisms supporting complex reasoning and adaptive decision-making. Parallel to Sternberg (2019), it can be stated that, together, they produce adaptive intelligence—a balanced use of intellect for collective benefit. Within sustainability contexts, this synergy enables individuals to critically interpret environmental challenges, formulate ethical judgments, and persist in sustainable behavior despite obstacles. Empirical findings confirm this dynamic relationship between these two constructs. Students with stronger critical thinking skills demonstrate more nuanced environmental reasoning and pro-sustainability attitudes (Sarıçam & Şahin, 2015). Self-regulation, similarly, predicts sustained engagement in eco-friendly behaviors and resilience in moral decision-making (Joukova et al., 2020; Morosanova et al., 2018). For gifted students, both skills are typically more developed (Preckel et al., 2020; Zimmerman and Martinez-Pons, 1990), suggesting that they may act as internal drivers of sustainable-living awareness and ethical commitment.

From a developmental standpoint, these constructs form an substantial psychological infrastructure of sustainable-living awareness. Sustainable-living awareness involves not only knowledge of environmental systems but also moral understanding and self-directed action. Critical thinking informs what individuals know and how they judge ethical consequences; self-regulation determines whether they act on that judgment. As Sternberg (2024b) emphasizes, intelligence achieves meaning only when it is governed by wisdom and sustained through moral self-regulation.

### 1.3 Integrative conceptual framework: giftedness, critical thinking, self-regulation, and sustainable-living awareness

This study conceptualizes transformational giftedness as a developmental system in which critical thinking and self-regulation function as psychosocial catalysts linking potential to sustainable-living awareness. In Sternberg (2024a) model, giftedness becomes transformational when intelligence and motivation are governed by ethical reasoning and self-control which are the forms of adaptive intelligence directed toward the common good. Critical thinking provides the cognitive foundation: analytical and reflective reasoning enables students to interpret complex and intricate environmental issues and evaluate moral consequences (Sarıçam & Şahin, 2015). Gifted students’ advanced reasoning and open-mindedness foster systems understanding and ethical discernment (Kuusisto and Tirri, 2015; Tolppanen et al., 2023). Self-regulation may support the motivation, translating judgment into sustained ethical behavior through goal setting, monitoring, and adaptive control. Empirical work shows that self-regulation predicts environmental engagement and mediates the link between ability and performance (Joukova et al., 2020; Morosanova et al., 2018). When combined, these mechanisms may operate synergistically: critical thinking directs ethical evaluation, while self-regulation sustains responsible action. Their interaction produces the psychosocial profile Sternberg (2019, 2024a) describes as transformational giftedness. Consequently, gifted students—with higher cognitive and regulatory capacities—are expected to display stronger sustainable-living awareness than their non-gifted peers.

Recent research further suggests that gifted students may develop deeper and more interconnected understandings of sustainability-related concepts when engaged in constructivist and participatory learning environments. For example, Bezen and Tanriöver (2026) demonstrated that game-based learning structured around the 5E model significantly enhanced gifted physics students’ conceptual understanding of the Sustainable Development Goals (SDGs), enabling students to produce more systemic, relational, and solution-oriented interpretations of sustainability issues. These findings support the argument that gifted students possess strong cognitive capacities for integrating complex socio-environmental concepts when appropriate pedagogical conditions are provided.

This framework also presents a justification for this study for a comparative design (gifted vs. non-gifted) as a developmental contrast. Before testing causal pathways, it is necessary to establish structural differences in these mechanisms. In this respect, Turkey’s Science and Arts Centers (SACs) provide an ideal context, emphasizing inquiry, autonomy, and social responsibility (Erol et al., 2023; MoNE, 2013; Sarıtaş et al., 2019; Usal and Buyurgan, 2022) conditions that mirror the psychosocial scaffolds Sternberg (2024a) associates with the transition from potential to transformational giftedness. Given that, the research questions of the study are stated below.

**Researh Question 1:** Do gifted students demonstrate higher levels of critical thinking, self-regulation, and sustainable-living awareness than non-gifted students?

**Researh Question 2.** To what extent do critical thinking and self-regulation predict sustainable-living awareness within gifted and non-gifted groups?

This study addresses three major research gaps identified by reviewers and prior literature. First, it establishes a clear problem statement by linking sustainable-living awareness to measurable psychological mechanisms rather than descriptive associations. Second, it grounds the analysis in Sternberg (2024a) integrative model of giftedness, bridging educational psychology and sustainability theory. Finally, it provides an empirically testable framework explaining how and why gifted students exhibit heightened sustainable-living awareness. By synthesizing Sternberg’s (2020, 2024a) notion of wisdom-based giftedness with ESD’s competence approach, this study repositions gifted education as a moral enterprise—one that fosters adaptive intelligence and ethical citizenship. Critical thinking and self-regulation emerge not as static traits but as developable capacities essential for wise, sustainable action. In practical terms, findings will inform differentiated education strategies that cultivate these competencies of gifted students for transformative potential. Ultimately, nurturing transformational giftedness serves both educational equity and global sustainability by developing leaders who, according to Sternberg (2024a), use their intelligence wisely, ethically, and for the common good.

## 2 Materials and methods

### 2.1 Participants

The study sample consisted of 266 middle school students (148 non-gifted, 118 gifted students). The non-gifted students included 64 males and 84 females (44 5th grade, 30 6th grade, 42 7th grade, 32 8th grade), while the gifted students included 58 males and 60 females (25 5th grade, 30 6th grade, 28 7th grade, 35 8th grade). Participants were selected through convenience sampling based on accessibility and willingness to participate. The adequacy of the questionnaire sample was evaluated using G*Power software. The analysis determined that a minimum of 251 participants was required to achieve a statistical power of 0.99. The actual sample size surpassed this threshold, thereby confirming its adequacy.

The gifted student population comprised individuals enrolled in Science and Arts Centers (SACs), specialized pull-out enrichment programs designed for gifted students. The study focuses on SACs because they are the primary formal institutions in Turkey specifically designed and mandated by the Ministry of National Education (MoNE) to provide supplemental education tailored to the unique needs of identified gifted students *outside* the regular school environment (Ministry of National Education-MoNE, 2013). They are the widespread, MoNE-operated institutions where identified gifted students receive their supplemental, ability-specific education nationwide, and they utilize standardized identification criteria (intelligence tests, talent assessments), adopt a multidisciplinary approach, and operate through a nationwide network (Epçaçan et al., 2020; Erol et al., 2023). There are over 300 SACs in Turkey, at least one in each province, and over 90,000 students in these schools (MoNE, 2024). All in all, SACs are purpose-built, nationally recognized, and internationally acclaimed institutions specifically designed for gifted education in Turkey (Sarıtaş et al., 2019). SACs are explicitly designed to offer differentiated, flexible, and comprehensive pull-out programs because gifted students have unique needs that regular schools often cannot meet. Therefore, the selection of SACs as the sample source is not only justified but also optimally suited for our research questions (Törün and Köksoy, 2023). The rationale is further supported by the literature review, which shows that previous Turkish research on gifted students’ perceptions frequently used SACs as the sample source (Epçaçan et al., 2020; Erol et al., 2023; Usal and Buyurgan, 2022).

These gifted students simultaneously attend mainstream schools. To ensure comparability in socioeconomic background, the non-gifted participants were selected from the same five general education schools attended by the identified gifted students. SACs are publicly funded pull-out programs that provide after-school enrichment opportunities for gifted students at the primary, middle, and high school levels. These programs prioritize advanced learning experiences and ability-based grouping (Sak, 2012). Admission to SACs follows a multi-stage selection process, which includes teacher nominations, group and individual intelligence assessments, and committee evaluations in specialized talent domains such as intellectual ability, visual arts, and music. The SAC curriculum is organized into five sequential phases: orientation, support, talent discovery, talent development, and project engagement, with a strong focus on project-based and problem-based learning (Güçyeter et al., 2017). Giftedness identification within SACs is conducted using the Anatolian-Sak Intelligence Scale (ASIS), an individually administered intelligence test designed for Turkish-speaking children aged 4 to 12 (Sözel et al., 2018). Developed based on the Cattell–Horn–Carroll intelligence model, ASIS was formed in Turkey in 2016 and comprises 256 items distributed across seven subtests and three cognitive domains. Its reliability and validity have been established through exploratory and confirmatory factor analyses (Arslan and Sak, 2023). Table 1 presents the demographic data of participants.

**Table 1:** Demographic data.

	Variables	Category	Count	Percent
Non-Gifted	Gender	Male	64	43.2
		Female	84	56.7
	Grade	5th	44	29.7
		6th	30	20.3
		7th	42	28.4
		8th	32	21.6
Gifted	Gender	Male	58	49.1
		Female	60	50.9
	Grade	5th	25	21.2
		6th	30	25.4
		7th	28	23.7
		8th	35	29.7

### 2.2 Measures

#### 2.2.1 Critical thinking disposition scale

The Critical Thinking Disposition Scale (CT; Döner and Demi̇r, 2021), consists of 21 items and operates across three dimensions. The scale employs a five-point Likert format, where responses range from 1 (“never”) to 5 (“always”). An example item from the scale is as follows: ‘I am able to justify the correctness of the solution I developed for a problem.’ When developing the scale, the researchers drew on Facione et al. (1995) in accordance with Turkish culture. It has been reported that the scale was tested by applying it to 503 middle school students for the validity and reliability study. The internal consistency coefficient (Cronbach’s alpha) for the instrument has been reported as 0.75, indicating a satisfactory level of reliability. Confirmatory factor analysis indicated a statistically significant chi-square and favorable fit across standard indices, and the three-factor solution accounted for a meaningful portion of the variance; together these results support an acceptable model fit and the scale’s construct validity. The total scale demonstrated a reliability coefficient of 0.85, while the subscales yielded values of 0.77 for dialectic thinking, 0.83 for disposition, and 0.75 for analysis in this study. Moreover, the scale has been employed in various prior studies, with its validity and reliability supported by empirical evidence (Öcal and Bahar, 2024).

#### 2.2.2 Perceived self-regulation scale

The Perceived Self-Regulation Scale (SR; Arslan and Gelişli, 2015), consists of 16 items and operates across two dimensions. The scale employs a five-point Likert format, where responses range from 1 (“never”) to 5 (“always”). Here is an example of an item from the scale: “When learning a subject, I develop different approaches to solve the problems I encounter.” The scale, developed in accordance with Turkish culture, is theoretically based on Vygotsky’s approaches (Vygotsky, 1978). It has been reported that the scale was tested by applying it to 604 middle school students for the validity and reliability study. The internal consistency coefficient (Cronbach’s alpha) for the instrument has been reported as 0.90, indicating a high level of reliability. Confirmatory factor analysis showed a statistically significant chi-square and favorable fit across conventional indices, and the two-factor solution accounted for the majority of the total variance. Therefore, the scale can be considered valid for use. The overall scale demonstrated a reliability coefficient of 0.77, while the subscales yielded values of 0.76 for openness and 0.70 for pursuit in this study. Additionally, the scale has been previously utilized in numerous studies, and its validity and reliability have been empirically tested and confirmed (Ateş and Sağar, 2024). The scale has also been employed in recent Turkish educational research with acceptable reliability and validity indices (e.g., Kuloğlu and Orhan, 2024).

#### 2.2.3 Sustainable-living awareness scale

The sustainable-living awareness Scale (SLA; Akgül and Aydoğdu, 2020), consists of 20 items and operates across three dimensions. The scale employs a three-point Likert format, where responses range from 1 (“disagree”) to 3 (“agree”). An example item from the scale is as follows: “The current rapid population growth has no impact on the consumption of natural resources.” The researchers theoretically grounded the scale development process in the work of Holmberg, and Sandbrook (2019). It has been reported that the scale was tested by applying it to 319 middle school students for the validity and reliability study. The internal consistency coefficient (Cronbach’s alpha) for the instrument has been reported as 0.77, indicating a satisfactory level of reliability. Confirmatory factor analysis indicated a statistically significant chi-square and satisfactory fit across conventional indices; the three-factor solution accounted for a meaningful portion of the variance, supporting acceptable model fit and construct validity. In the current study, Cronbach’s alpha internal consistency coefficients were obtained as 0.75 for the total scale, while the subscales yielded values of 0.74 for society, 0.71 for environment and 0.70 for economy in this study. Furthermore, the scale has been applied in several previous studies, and its validity and reliability have been substantiated through empirical findings (Durak and Karayağız Muslu, 2023).

### 2.3 Procedure and data collection

Data were collected from SACs and public schools using identical procedures for gifted and non-gifted students between 15 September and 31 October 2024. School directors and teachers were briefed, and written informed consent was obtained from parents and children. The study received ethical approval from the Harran University Social Science Ethics Committee (approval no. E-76244175-050.04-421463).

### 2.4 Statistical analysis

Before conducting the statistical analysis to address the study’s objectives, the data underwent organization and assumption testing. These preliminary evaluations included an analysis of missing values, detection of extreme values, and assessments of normality and linearity. The first step involved examining missing data, which identified nine missing values that were subsequently excluded. For extreme value detection, variable scores were standardized into z-scores to assess their deviation from the mean. As no z-scores exceeded the ±3 threshold, no outliers were identified. Normality was evaluated through skewness and kurtosis values, both of which fell within the recommended range of −1.5 to +1.5, indicating no substantial deviation from a normal distribution. Furthermore, the Kolmogorov–Smirnov test yielded *p*-values greater than 0.05 for both pre-test and post-test data (see Table 2), further supporting the assumption of normality. These results align with the guidelines established by Tabachnick and Fidell (2007).

**Table 2:** Results of normal distribution tests.

		Skewness		Kurtosis		Test of normality (Kolmogorov–Smirnov)			Levene’s test	Box’s M test
		Statistic	Std. Error	Statistic	Std. Error	Statistic	*df*	Sig.	Sig.	Sig.
CT	Gifted	−0.052	0.223	−0.621	0.442	0.055	118	0.200	0.064	
	Non-gifted	0.212	0.200	−0.326	0.400	0.091	148	0.104		
SR	Gifted	−0.044	0.223	−0.213	0.442	0.052	118	0.200	0.069	0.032
	Non-gifted	−0.010	0.200	−0.274	0.400	0.050	148	0.200		
SLA	Gifted	−0.769	0.223	0.849	0.442	0.173	118	0.200	0.067	
	Non-gifted	0.210	0.200	−0.210	0.400	0.084	148	0.113		

In addition, assumptions related to homogeneity of variance, homogeneity of covariance matrices, and multicollinearity were evaluated before conducting the primary analyses. Multicollinearity assumptions were examined using tolerance and variance inflation factor (VIF) values. All tolerance values exceeded 0.20 (minimum tolerance = 0.764) and all VIF values were below 5 (maximum VIF = 1.308), indicating no multicollinearity concerns. Following assumption checks, descriptive statistics, correlational analyses, group comparisons, and multiple regression analyses were conducted to address the research questions. Effect sizes were interpreted according to established criteria and interpreted according to Cohen’s (1988) guidelines. Assumptions of Multivariate Analysis of Variance (MANOVA) were evaluated prior to the analyses. Box’s M test indicated that the assumption of homogeneity of covariance matrices was satisfied (*p* > 0.001). Levene’s tests also demonstrated acceptable homogeneity of variances across dependent variables. Descriptive statistics for the study variables are provided in Table 2.

After completing the analyses for missing values, extreme values, normality distribution, and linearity, the data analysis process commenced. To address Research Question 1 (whether gifted and non-gifted students differ on critical thinking, self-regulation and sustainable-living awareness), group differences for each outcome variable were examined with independent-samples t-tests. Student’s t-tests were used to compare differences between two groups based on their distribution characteristics. To assess relationships between variables, Pearson’s correlation test was applied to calculate correlation coefficients and significance for normally distributed parameters, while Spearman’s correlation test was used for non-normally distributed parameters. Because the dependent variables form a set of related outcomes, MANOVA was also performed to evaluate the omnibus effects of Group (gifted vs. non-gifted), Gender (male vs. female), and the Group × Gender interaction on the combined dependent variables. Statistical significance was set at *p* < 0.05. Finally, to address Research Question 2 (the extent to which critical thinking and self-regulation predict sustainable-living awareness within each subgroup), two separate multiple linear regression models were estimated — one for the gifted sample and one for the non-gifted sample — with sustainable-living awareness as the dependent variable and critical thinking and self-regulation as predictors.

Multiple linear regression is a standard and appropriate method for modeling a continuous outcome as a function of multiple predictors (Best and Wolf, 2015). In this study, sustainable-living awareness is assessed on a continuous scale, with critical thinking and self-regulation serving as continuous predictor variables. This approach fits a linear equation to relate the dependent variable to the explanatory variables, revealing each predictor’s contribution while holding others constant. It is well-established under verified assumptions, including approximate linearity between predictors and the outcome, normally distributed residuals, and absence of perfect multicollinearity. The model’s *R^2^* and adjusted *R^2^* quantify the proportion of variance in environmental awareness explained by the predictors collectively, providing an unbiased estimate. Regression coefficients (*β*) estimate the expected change in the outcome per unit change in each predictor, controlling for the other, with t-tests evaluating statistical significance (Best and Wolf, 2015). This framework directly addresses the research question by quantifying the strength and significance of each independent variable’s predictive effect on environmental awareness, while accounting for the other.

Finally, alternative methods were considered but deemed less suitable. Logistic or ordinal regression is appropriate for categorical outcomes, not our continuous one. Structural equation modeling (SEM) could model these relationships but offers equivalent estimates to ordinary least squares (OLS) regression with unnecessary complexity, absent latent variables or intricate paths. Simple correlations or bivariate analyses fail to account for multiple predictors’ simultaneous effects. In summary, multiple linear regression is justified as it aligns with our variables’ scale and nature, directly addresses predictive questions, and relies on established statistical theory (Best and Wolf, 2015).

## 3 Results

An examination of critical thinking disposition, self-regulation, and sustainable-living awareness scores revealed that within each group, males and females reported similar mean scores (See Table 3). Independent samples t-tests were conducted to determine whether there were significant gender differences within the gifted and non-gifted groups. The results indicated no significant differences between males and females within each group across all factors. Specifically, among gifted students, no significant gender differences were observed in self-regulation (*t*[116] = 0.103, *p* > 0.05), critical thinking disposition (*t*[116] = −1.057, *p* > 0.05), or sustainable-living awareness (*t*[116] = −2.187, *p* > 0.05). Similarly, among non-gifted students, males and females showed no significant differences in self-regulation [t(146) = −1.167, *p* > 0.05], critical thinking disposition (t[146] = −0.173, p > 0.05), or sustainable-living awareness (*t* [146] = 0.223, *p* > 0.05). These findings indicate that while scores varied between the gifted and non-gifted groups, gender differences within each group were not statistically significant.

**Table 3:** Independent-samples *t*-test results for gender differences in critical thinking disposition, self-regulation, and sustainable-living awareness within gifted and non-gifted groups.

	Gifted				Non-gifted					
	Male (*n =* 58)	Female (*n =* 60)	*t* or *z*	*df*	*p*	Male (*n =* 64)	Female (*n =* 84)	*t* or *z*	*df*	*p*
CT^1^	4.14	4.23	−1.057	116	0.292	3.55	3.57	−0.173	146	0.863
CTdialect	4.30	4.36	−0.655	116	0.514	3.81	3.86	−0.565	146	0.573
CTdisposition	3.66	4.00	−1.796	116	0.075	3.25	3.18	0.382	146	0.703
CTanalysis	4.15	4.11	0.402	116	0.689	3.18	3.17	0.032	146	0.975
SR^2^	3.90	3.89	0.103	116	0.918	3.52	3.63	−1.167	146	0.245
SR openness	3.98	4.04	−0.738	116	0.462	3.55	3.71	−1.527	146	0.129
SR pursuit	3.83	3.75	0.639	116	0.524	3.48	3.54	−0.545	146	0.586
SLA^3^	2.52	2.61	−2.187	116	0.081	2.09	2.08	0.223	146	0.824
SLAsociety	2.50	2.56	−0.977	116	0.331	1.98	2.00	−0.377	146	0.707
SLAenvironment	2.54	2.68	−2.596	116	0.071	2.19	2.14	0.704	146	0.482
SLAeconomy	2.52	2.61	−1.276	116	0.204	2.11	2.10	0.088	146	0.930

^1^CT, Critical Thinking Disposition. ^2^SR, Self-Regulation. ^3^SLA, Sustainable-Living Awareness.

To examine the potential effects of giftedness and gender on self-regulation, critical thinking, and self-awareness, a 2 × 2 (giftedness × gender) MANOVA was conducted on both the overall scales and their respective dimensions. The results demonstrated a significant effect of giftedness; however, no significant effect was observed for gender. Specifically, a main effect was found for giftedness (Wilk’s Lambda = 0.36, F[8, 255] = 56.00, *p* < 0.001, η^2^^p^ = 0.64], whereas gender did not have a significant impact [Wilk’s Lambda = 0.97], F[8, 255] = 1.04, *p* > 0 0.001, η^2^^p^ = 0.03). Consequently, follow-up analyses of variance (ANOVAs) were performed to further investigate differences between gifted and non-gifted students across the scales and their dimensions. The findings indicated that gifted students significantly outperformed their non-gifted peers in self-regulation (F[1, 264] = 23.74, *p* < 0.001, η^2^ = 0.05), critical thinking (F[1, 264] = 93.19, *p* < 0.001, η^2^ = 0.19), and sustainable-living awareness (F[1, 264] = 347.83, *p* < 0 0.001, η^2^ = 0.12). Similar significance levels were also reported for the sub-dimensions (see Table 4). Taken together, these results validate the hypotheses of this study by revealing that gifted students exhibit significantly distinct levels of critical thinking, self-regulation, and sustainable-living awareness compared to their non-gifted peers.

**Table 4:** One-Way ANOVA results for differences in critical thinking disposition, self-regulation, and sustainable-living awareness by giftedness status.

Variables	Gifted		Nongifted		MS	F	*p*	*η* ^2^
	M	SD	M	SD				
CT	4.19	0.44	3.56	0.58	25.75	93.19	0.000	0.19
CTdialect	4.33	0.45	3.84	0.59	15.88	56.85	0.000	0.12
CTdisposition	3.83	1.04	3.21	1.12	25.50	21.55	0.000	0.19
CTanalysis	4.13	0.56	3.18	0.82	59.43	115.40	0.000	0.45
SR	3.90	0.49	3.58	0.56	6.71	23.74	0.000	0.05
SRopenness	4.01	0.45	3.64	0.63	8.65	28.05	0.000	0.06
SRpursuit	3.79	0.68	3.51	0.66	5.02	11.22	0.000	0.03
SLA	2.58	0.23	2.08	0.19	15.53	347.83	0.000	0.12
SLAsociety	2.53	0.32	1.99	0.33	18.95	173.85	0.000	0.14
SLAenvironment	2.61	0.29	2.16	0.35	13.25	125.74	0.000	0.10
SLAeconomy	2.57	0.35	2.11	0.37	13.69	105.40	0.000	0.10

In the gifted group, a significant correlation was observed among self-regulation skills, critical thinking disposition, and sustainable-living awareness levels (See Table 5). Similarly, in the non-gifted group, these skills were also meaningfully correlated; however, the strength of these relationships appeared to be weaker compared to the gifted group.

**Table 5:** Pearson correlation coefficients among critical thinking disposition, self-regulation, and sustainable-living awareness in gifted students.

	Skill	CT	SR	SLA
Gifted	SR		1	
	CT	1	0.640**	
	SLA	0.485**	0.514**	1
Non-gifted	SR		1	
	CT	1	0.548**	
	SLA	0.199*	0.238**	1

**Correlation is significant at the 0.01 level (2-tailed). *Correlation is significant at the 0.05 level (2-tailed).

A multiple regression analysis was conducted to examine the extent to which SR and critical thinking CT predict sustainable-living awareness in gifted and non-gifted groups (See Table 6). For the gifted students, the regression model was statistically significant, *F*(2, 115) = 25.272, p < 0.001, explaining approximately 30.5% of the variance in SLA (*R*^2^ = 0.0305, Adjusted *R*^2^ = 0.293). The Durbin-Watson statistic (1.768) indicated that there was no serious autocorrelation in the residuals. The unstandardized and standardized regression coefficients, along with their significance levels, are presented in Table 6. The results showed that both SR (*β* = 0.344, *p* = 0.001) and CT (*β* = 0.265, *p* = 0.010) were significant positive predictors of SLA. Specifically, for each one-unit increase in SR, SLA increased by 0.152 units (*B* = 0.152, *p* = 0.001), while for each one-unit increase in CT, SLA increased by 0.130 units (*B* = 0.130, *p* = 0.010). The collinearity statistics indicated no multicollinearity concerns, with tolerance values of 0.590 and variance inflation factors (VIF) of 1.695 for both predictors. These findings suggest that both critical thinking and self-regulation contribute significantly to sustainable-living awareness. Afterwards, another model was tested to capture whether awareness is developmental and whether development (SR × course and CT × course) modulates the effects of critical thinking disposition and self-regulation for gifted students. Results indicated that model was statistically significant, *F* (2, 115) = 5.634, *p* < 0.001, explaining approximately 16.6% variance in SLA (*R*^2^ = 0.166, Adjusted *R*^2^ = 0.137). The Durbin-Watson statistic (1.894) indicated that there was no serious autocorrelation in the residuals. The findings revealed that both SR × course (*β* = 0.059, *p* = 0.120) and CT × course (*β* = −0.042, *p* = 0.229) were not significant predictors of SLA.

**Table 6:** Multiple regression analysis predicting sustainable-living awareness.

Model	Predictor	B	SE	*β*	*t*	*p*	Significance level	Tolerance	VIF
Gifted	(Constant)	1.490	0.165	–	9.009	<0.001	1.16–1.81	–	–
	CT	0.130	0.050	0.265	2.622	0.010	0.03–0.22	0.59	1.69
	SR	0.152	0.045	0.344	3.397	0.001	0.06–0.24	0.59	1.69
Non-gifted	(Constant)	1.740	0.111	–	15.665	<0.001	1.52–1.95	–	–
	CT	0.032	0.032	0.098	1.015	0.312	−0.03–0.09	0.69	1.43
	SR	0.063	0.033	0.185	1.921	0.050	−0.00–0.12	0.69	1.43

*B, unstandardized regression coefficient; SE, standard error; *β*, standardized regression coefficient; t, t statistic; *p*, significance level; VIF, variance inflation factor.

For the non-gifted students, the regression model was statistically significant, *F*(2, 145) = 4.902, *p* = 0.009, but explained only 6.3% of the variance in SLA (*R*^2^ = 0.063, Adjusted *R*^2^ = 0.050). The Durbin-Watson statistic (1.816) suggested no serious autocorrelation issues in the residuals. The regression coefficients are presented in Table 6. The results indicate that SR was a marginally significant positive predictor of SLA (*β* = 0.185, *p* = 0.057). CT, however, was not a significant predictor of SLA (*β* = 0.098, *p* = 0.312). The collinearity statistics (Tolerance = 0.699, VIF = 1.430 for both predictors) suggested that multicollinearity was not a concern. These findings indicate that SR and CT strongly predict SLA in gifted students, whereas these variables exhibit lower explanatory power in non-gifted students. Notably, SR emerged as a stronger predictor among gifted students, while its significance in non-gifted students remained at the threshold level. These results suggest that gifted students may utilize their cognitive and self-regulation skills more effectively in the process of developing sustainable-living awareness. In summary, these results confirm the study’s final hypothesis that critical thinking and self-regulation serve as positive and significant predictors of environmental awareness for gifted students. Afterwards, another model was tested to capture whether awareness is developmental and whether development (SR × course and CT × course) modulates the effects of critical thinking and self-regulation for non-gifted students. Findings showed that model was not statistically significant F(2, 145) = 3.042, *p* > 0.001 (*p* = 0.031), explained only 6% of the variance in SLA (*R*^2^ = 0.060, Adjusted *R*^2^ = 0.050). The results indicated that both SR × course (*β* = 0.023, *p* = 0.082) and CT × course (*β* = 0.011, *p* = 0 0.406) were not significant predictors of SLA for non-gifted students.

## 4 Discussion

This study investigated whether gifted middle-school students demonstrate higher levels of critical thinking, self-regulation, and sustainable-living awareness than their non-gifted peers, and whether the first two variables predict sustainable-living awareness across groups. Guided by Sternberg’s (2020, 2024a) framework of transformational giftedness, the findings illuminate how giftedness functions not merely as elevated intellectual potential but as a psychosocial capacity for adaptive, ethical, and prosocial action.

For the first research question, gifted students scored significantly higher in critical thinking and self-regulation than their non-gifted peers. This finding aligns with classic work on metacognitive awareness and self-regulated learning (Risemberg and Zimmerman, 1992; Zimmerman and Martinez-Pons, 1990) and with contemporary perspectives portraying gifted students as reflective and autonomous thinkers (Shavinina, 2012). Regression analyses further addressed the second research question, indicating that both critical thinking and self-regulation significantly predicted sustainable-living awareness and that their interaction strengthened this relationship. The present findings are also consistent with prior research emphasizing the interconnected nature of higher-order thinking processes among gifted students. Boran and Karakuş (2022) reported that metacognitive awareness and critical thinking disposition jointly contributed to gifted students’ perceived problem-solving skills, highlighting the integrative role of reflective and analytical thinking processes in complex cognitive functioning. These outcomes echo Sternberg (2024b) proposition that adaptive intelligence emerges from the integration of analytical reasoning, self-governance, and moral intent. Students who can think critically about complex sustainability dilemmas and regulate their behavior toward ethical goals are more likely to demonstrate sustainability-oriented awareness and action. Hence, the present study empirically operationalizes the psychosocial mechanism underlying transformational giftedness: critical thinking and self-regulation jointly transform potential into socially responsible awareness.

Our results converge with the findings of Tolppanen et al. (2023), who reported that academically gifted students exhibit stronger climate competencies than their non-gifted peers and that those studying in selective school contexts display greater willingness for societal (group) action, supported by higher perceptions of teacher support, student agency, and future-career relevance. Together, these results suggest that individual mechanisms (critical thinking and self-regulation) and contextual affordances, such as supportive school climates, constitute complementary pathways for narrowing the awareness–action gap. Although gifted students demonstrated higher levels of sustainable-living awareness, prior research cautions that awareness alone does not automatically yield consistent pro-environmental behavior—a phenomenon known as the knowledge–action gap (Mutlu & Nacaroğlu, 2019). This gap implies the disjunction between knowing about sustainability and acting upon that knowledge. The current findings indicate that bridging this gap depends on activating self-regulatory and motivational resources. In Sternberg (2024a) terms, this transformation marks the developmental progression from intelligence to wisdom that is the practical application of ability for the common good. Consistent with this interpretation, Koçulu and Topçu (2024) emphasize that sustainability education must move beyond declarative knowledge toward participatory engagement through “structured inquiry, collaborative problem-solving, and reflection cycles that transform understanding into behavioral intention.” Embedding such iterative pedagogical designs within gifted education can help close the knowledge–action gap by allowing students to exercise moral reasoning, self-regulation, and ecological agency in authentic contexts.

Accordingly, narrowing the awareness–action gap requires self-regulatory scaffolding (goal setting, monitoring, and reflection) combined with participatory learning that cultivates agency and shared norms for action. Echoing these implications, Tolppanen et al. (2023) found that perceived teacher support and student agency were associated with greater willingness for group-level environmental engagement. The present findings are consistent with recent research suggesting that gifted students develop more holistic and solution-oriented understandings of sustainability issues when engaged in constructivist learning environments (Bezen and Tanriöver, 2026). They also support evidence indicating that critical thinking and self-regulation function as mutually reinforcing cognitive processes during complex learning and decision-making tasks, highlighting the integrative role of reflective cognition in adolescent development (Kuloğlu & Orhan, 2024). These similar findings reinforce the interpretation that self-regulation and contextual supports jointly convert sustainable-living awareness into sustained, prosocial behavior in action.

Transformational giftedness is inherently shaped by sociocultural contexts. Turkey’s Science and Arts Centers (SACs) embody an educational ecosystem emphasizing inquiry, autonomy, and social responsibility (Erol et al., 2023; MoNE, 2013; Sarıtaş et al., 2019). These features mirror Sternberg (2024b) psychosocial scaffolding for adaptive intelligence: environments that foster moral reasoning, reflection, and prosocial application of ability. However, cultural values influence how such abilities manifest. In collectivist contexts, moral responsibility and community welfare often take precedence over individual achievement (Kuusisto & Tirri, 2015). Thus, Turkish gifted students’ sustainability orientation may emerge through collaborative and civic engagement rather than through purely individual problem-solving, distinguishing them from Western conceptions of giftedness centered on personal innovation. This sociocultural mediation underscores the importance of contextualizing transformational giftedness: its behavioral expressions and actions (and their impact on sustainability) depend on how societies define ethical contribution and civic responsibility.

Additionally, focusing on gifted students raises a critical ethical tension: how to reconcile differentiated and enriched education with inclusive sustainability goals. Rather than perpetuating elitism, this research aligns with Sternberg (2024a) argument that gifted education should serve societal advancement. The aim is to understand mechanisms (critical thinking and self-regulation in this study) that can later be generalized to all students. From an inclusion standpoint, empowering all students with these psychosocial competencies promotes equity. As Sternberg notes, “wisdom is teachable”; thus, fostering ethical reasoning and self-governance need not be confined to high-ability groups. Gifted programs can function as developmental laboratories for scalable pedagogical innovations, helping to democratize transformational learning across diverse populations.

## 5 Implications

The findings of this study have several important implications for educational practice, policy, and future research. From an educational perspective, sustainability education should move beyond the transmission of environmental knowledge toward cultivating the psychosocial capacities that enable students to think critically and act responsibly. Embedding critical thinking and self-regulation within pedagogy through, for example, inquiry-based, interdisciplinary, and ethically oriented projects, can foster students’ ability to navigate complexity and moral issues related to environmental problems. In gifted education specifically, programs should evolve and move from traditional enrichment toward cultivating ethical leadership for sustainability, directly operationalizing Sternberg (2024a) proposal for wisdom-based education. Recent evidence also suggests that constructivist and participatory pedagogies, including game-based sustainability education, may help gifted students develop more integrated understandings of environmental and social challenges (Bezen and Tanriöver, 2026). Accordingly, gifted education programs may benefit from incorporating interdisciplinary and experiential sustainability-oriented learning environments. Schools should also provide authentic learning environments where students can translate awareness into concrete action. Such experiential contexts strengthen both self-regulatory control and moral competence, bridging the persistent gap between sustainability knowledge and behavior. At the policy and systemic level, promoting psychosocial skill development across all student profiles is crucial for fostering inclusive and responsible citizenship. Critical thinking and self-regulation should be viewed not as privileges of giftedness but as foundational capacities for all students. Finally, the study carries meaningful implications for research and theory. By empirically substantiating the relationship between adaptive intelligence and sustainable-living awareness, the findings extend Sternberg’s theoretical model, highlighting moral motivation as a potential mediating mechanism between cognition and ethical behavior. Future studies should employ comparative and longitudinal designs to examine how these psychosocial processes develop across time and cultural contexts, thereby enhancing external validity.

## 6 Limitations

Despite its contributions, several limitations warrant consideration. First, the study relied on self-reported measures, which may be susceptible to social-desirability or metacognitive bias. Future research should employ behavioral or performance-based assessments, such as digital trace data, peer evaluations, or authentic environmental tasks, to capture enacted sustainability competence. Second, although the study’s cross-sectional design limits causal inference, it included controls for age and grade-level differences, mitigating developmental bias. Longitudinal designs would allow observation of how critical thinking and self-regulation evolve and interact over time to predict sustained engagement. Third, the research context was culturally specific to Turkey’s SAC system. Educational policies, cultural norms, and sustainability discourses vary globally; cross-national comparisons would illuminate how sociocultural mediation shapes transformational giftedness. Finally, the sample was restricted to middle-school students. Future longitudinal studies should follow students into secondary and higher education to explore the developmental stability of these mechanisms and their translation into civic or professional sustainability engagement.

## 7 Conclusion

The present study confirms that gifted middle-school students display significantly higher levels of critical thinking, self-regulation, and sustainable-living awareness than non-gifted peers. More importantly, it reveals that critical thinking and self-regulation are not parallel predictors but interacting mechanisms underpinning sustainable-living awareness. Drawing upon Sternberg’s (2020, 2024a) vision of transformational giftedness, the findings show that when intellectual ability is coupled with self-governance and ethical intent, it yields adaptive outcomes that contribute to sustainable societies. Giftedness, therefore, should be understood as the capacity to use one’s abilities for the common good and is a form of ethical intelligence that education can and should cultivate and foster. The study contributes conceptually by integrating psychological constructs (critical thinking, self-regulation) within sustainability education and empirically by demonstrating their predictive value in adolescence. It thus positions gifted students as developmental exemplars of how higher-order cognition and self-regulatory discipline translate into sustainability-oriented awareness and potential leadership.

## Data Availability

The raw data supporting the conclusions of this article will be made available by the authors, without undue reservation.
